# Supplementary material for: TAF15 amyloid filaments in frontotemporal lobar degeneration
Source: Nature. 2023 Dec 6;625(7994):345–51. doi: 10.1038/s41586-023-06801-2 (PMC10781619; doi:10.1038/s41586-023-06801-2)

nature portfolio

https://doi.org/10.1038/s41586-023-06801-2

Supplementary information

TAF15 amyloid filaments in frontotemporal lobar degeneration

In the format provided by the authors and unedited

Supplementary Figure 1: Uncropped images of immunoblots shown in this study

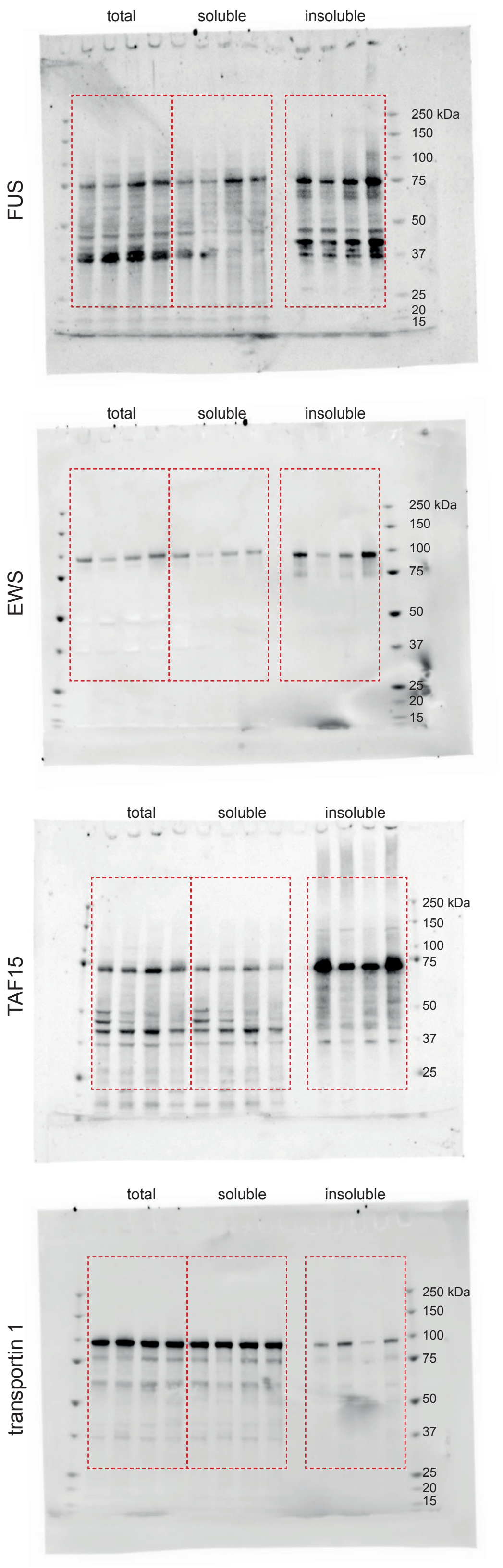

Supplement: Supplementary file 1 — Uncropped images of immunoblots shown in this study. [file 41586_2023_6801_MOESM1_ESM.pdf]
