# Supplementary material for: TAF15 amyloid filaments in frontotemporal lobar degeneration
Source: Nature. 2023 Dec 6;625(7994):345–51. doi: 10.1038/s41586-023-06801-2 (PMC10781619; doi:10.1038/s41586-023-06801-2)
Supplement: Supplementary file 2 — Reporting Summary [file 41586_2023_6801_MOESM2_ESM.pdf]

nature portfolio

Corresponding author(s): Benjamin Ryskeldi-Falcon

Last updated by author(s): Oct 18, 2023

Reporting Summary

Nature Portfolio wishes to improve the reproducibility of the work that we publish. This form provides structure for consistency and transparency in reporting. For further information on Nature Portfolio policies, see our Editorial Policies and the Editorial Policy Checklist.

Statistics

For all statistical analyses, confirm that the following items are present in the figure legend, table legend, main text, or Methods section.

n/a	Confirmed	
☐	☒	The exact sample size (*n*) for each experimental group/condition, given as a discrete number and unit of measurement
☐	☒	A statement on whether measurements were taken from distinct samples or whether the same sample was measured repeatedly
☒	☐	The statistical test(s) used AND whether they are one- or two-sided<br>*Only common tests should be described solely by name; describe more complex techniques in the Methods section.*
☒	☐	A description of all covariates tested
☒	☐	A description of any assumptions or corrections, such as tests of normality and adjustment for multiple comparisons
☐	☒	A full description of the statistical parameters including central tendency (e.g. means) or other basic estimates (e.g. regression coefficient) AND variation (e.g. standard deviation) or associated estimates of uncertainty (e.g. confidence intervals)
☒	☐	For null hypothesis testing, the test statistic (e.g. *F*, *t*, *r*) with confidence intervals, effect sizes, degrees of freedom and *P* value noted<br>*Give P values as exact values whenever suitable.*
☐	☒	For Bayesian analysis, information on the choice of priors and Markov chain Monte Carlo settings
☒	☐	For hierarchical and complex designs, identification of the appropriate level for tests and full reporting of outcomes
☒	☐	Estimates of effect sizes (e.g. Cohen's *d*, Pearson's *r*), indicating how they were calculated

Our web collection on statistics for biologists contains articles on many of the points above.

Software and code

Policy information about availability of computer code

Data collection	EPU 2.14
Data analysis	DIA-NN 1.8.1, RELION 4.0, CTFFIND 4.1, COOT 0.9.8.2, ISOLDE 1.5, REFMAC 5.8.0387, Servalcat 0.3.0, ChimeraX 1.5, MolProbity 4.5.2.

For manuscripts utilizing custom algorithms or software that are central to the research but not yet described in published literature, software must be made available to editors and reviewers. We strongly encourage code deposition in a community repository (e.g. GitHub). See the Nature Portfolio guidelines for submitting code & software for further information.

Data

Policy information about availability of data

All manuscripts must include a data availability statement. This statement should provide the following information, where applicable:

- Accession codes, unique identifiers, or web links for publicly available datasets
- A description of any restrictions on data availability
- For clinical datasets or third party data, please ensure that the statement adheres to our policy

Whole-exome data have been deposited in the National Institute on Ageing Alzheimer’s Disease Data Storage Site (NIAGADS), under accession code NG00107. Mass spectrometry data have been deposited to the Proteomics Identifications (PRIDE) database under accession code PXD044821. Cryo-EM datasets have been deposited to the Electron Microscopy Public Image Archive (EMPIAR) under accession codes EMPIAR-11735 (individual 1, prefrontal cortex); EMPIAR-11736 (individual 1, motor cortex); EMPIAR-11737 (individual 2 prefrontal and temporal cortex); EMPIAR-11738 (individual 3, prefrontal and temporal cortex);

EMPIAR-11739 (individual 4, prefrontal cortex); and EMPIAR-11740 (individual 4, brainstem). Cryo-EM maps have been deposited to the Electron Microscopy Data Bank (EMDB) under accession codes EMD-16999 and EMD-18236 (TAF15 filaments from prefrontal cortex and motor cortex, respectively, of individual 1); EMD-17022 (TAF15 filaments from prefrontal and temporal cortex of individual 2); EMD-17021 (TAF15 filaments from prefrontal and temporal cortex of individual 3); EMD-17020 and EMD-18227 (TAF15 filaments from prefrontal cortex and brainstem, respectively, of individual 4); EMD-17109 and EMD-18226 (Aβ42 filaments from prefrontal cortex and brainstem, respectively, of individual 4); EMD-18240 and EMD-18243 (singlet TMEM106B filaments from prefrontal cortex and brainstem, respectively, of individual 4); and EMD-18242 and EMD-18241 (doublet TMEM106B filaments from prefrontal cortex and brainstem, respectively, of individual 4). The atomic model for the TAF15 amyloid filaments has been deposited to the Protein Data Bank (PDB) under accession code 8ONS. The atomic models of singlet and doublet TMEM106B type I filaments are available at the PDB under accession codes 7qvc and 7qvf, respectively. The atomic model of Aβ42 type II filaments is available at the PDB under accession code 7q4m.

Human research participants

Policy information about studies involving human research participants and Sex and Gender in Research.

Reporting on sex and gender	1 female and 4 males.
Population characteristics	See Extended Data Table 1. Between 30 and 79 years-of-age. No neurodegenerative disease associated mutations. Clinical diagnoses of bvFTD and ALS. Neuropathological diagnosis of FTLD-FET.
Recruitment	Selected based on availability and neuropathological examination.
Ethics oversight	Human tissue samples were from the Brain Library of the Dementia Laboratory at Indiana University School of Medicine and the Queen Square Brain Bank for Neurological Disorders at UCL Queen Square Institute of Neurology. Their use in this study was approved by the ethical review processes at each institution.

Note that full information on the approval of the study protocol must also be provided in the manuscript.

Field-specific reporting

Please select the one below that is the best fit for your research. If you are not sure, read the appropriate sections before making your selection.

☒ Life sciences ☐ Behavioural & social sciences ☐ Ecological, evolutionary & environmental sciences

For a reference copy of the document with all sections, see nature.com/documents/nr-reporting-summary-flat.pdf

Life sciences study design

All studies must disclose on these points even when the disclosure is negative.

Sample size	Frontotemporal cortex from 4 individuals with FTLD-FET. Samples were chosen based on availability and neuropathological examination.
Data exclusions	Pre-established common image classification procedures (Scheres 2012. J. Struc. Biol. 180, 519-530) were employed to select particle images with the highest resolution content in the cryo-EM reconstruction process. Details of the number of selected images are given in Extended Data Table 2.
Replication	All attempts at replication were successful. Four independent biological repeats per experiment where representative data are shown, as described in the main text.
Randomization	Randomisation was not performed. As the samples were limited by brain availability, randomisation would not have reduced any bias in this study.
Blinding	The investigators were not blinded to allocation during experiments and outcome assessment. The perceived risk of detection/performance bias was deemed negligible.

Reporting for specific materials, systems and methods

We require information from authors about some types of materials, experimental systems and methods used in many studies. Here, indicate whether each material, system or method listed is relevant to your study. If you are not sure if a list item applies to your research, read the appropriate section before selecting a response.

Materials & experimental systems

n/a	Involved in the study
☐	☒ Antibodies
☒	☐ Eukaryotic cell lines
☒	☐ Palaeontology and archaeology
☒	☐ Animals and other organisms
☒	☐ Clinical data
☒	☐ Dual use research of concern

Methods

n/a	Involved in the study
☒	☐ ChIP-seq
☒	☐ Flow cytometry
☒	☐ MRI-based neuroimaging

Antibodies

Antibodies used	The primary antibodies used were anti-FUS (Proteintech 11570-1-AP), anti-TAF15 (Bethyl IHC-00094), anti-TAF15 (Bethyl A300-308A), anti-EWS (Santa Cruz sc-28327) and anti-transportin-1 (Abcam ab10303).
Validation	Antibody validation is presented in the manufacturers' datasheets, as well as in (Neumann et al. 2011. Brain 9, 2595–2609), (Brelstaff et al. 2011. Acta Neuropathol. 5, 591–600), (Neumann et al. 2012. Acta Neuropathol. 5, 705–716) and (Davidson et al. 2013. Neuropathol. Appl. Neurobiol. 2, 157–165).
